# *In Vivo* Accumulation of *Helicobacter pylori* Products, NOD1, Ubiquitinated Proteins and Proteasome in a Novel Cytoplasmic Structure

**DOI:** 10.1371/journal.pone.0009716

**Published:** 2010-03-16

**Authors:** Vittorio Necchi, Patrizia Sommi, Vittorio Ricci, Enrico Solcia

**Affiliations:** 1 Department of Human Pathology, University of Pavia Medical School, Pavia, Italy; 2 Department of Physiology, University of Pavia Medical School, Pavia, Italy; 3 Pathologic Anatomy Service, Fondazione IRCCS Policlinico San Matteo, Pavia, Italy; Charité-Universitätsmedizin Berlin, Germany

## Abstract

Cell internalization and intracellular fate of *H. pylori* products/virulence factors *in vivo* by human gastric epithelium, the main target of *H. pylori*-induced pathologies (i.e., peptic ulcer and cancer), are still largely unknown. Investigating gastric endoscopic biopsies from dyspeptic patients by means of ultrastructural immunocytochemistry, here we show that, in human superficial-foveolar epithelium and its metaplastic or dysplastic foci, *H. pylori* virulence factors accumulated in a discrete cytoplasmic structure characterized by 13-nm-thick cylindrical particles of regular punctate-linear substructure resembling the proteasome complex in size and structure. Inside this particle-rich cytoplasmic structure (PaCS) we observed colocalization of VacA, CagA, urease and outer membrane proteins with NOD1 receptor, ubiquitin-activating enzyme E1, polyubiquitinated proteins, proteasome components and potentially oncogenic proteins like SHP2 and ERKs in human gastric epithelium. By means of electron and confocal microscopy, we demonstrate that the *in vivo* findings were reproduced *in vitro* by incubating human epithelial cell lines with *H. pylori* products/virulence factors. PaCSs differed from VacA-induced vacuoles, phagosomes, aggresomes or related bodies. Our data suggest that PaCS is a novel, proteasome-enriched structure arising in ribosome-rich cytoplasm at sites of *H. pylori* products accumulation. As a site of selective concentration of bacterial virulence factors, the ubiquitin-proteasome system and interactive proteins, PaCS is likely to modulate immune-inflammatory and proliferative responses of the gastric epithelium of potential pathologic relevance.

## Introduction

Gastric superficial-foveolar epithelium is the main site of host/bacterial interaction in *H. pylori* infection. *H. pylori* contacts and adheres to the luminal surface of the epithelium, may enter intercellular lateral spaces and may even penetrate inside the cell cytoplasm [1], [2]. At this intracellular site, the bacteria are usually enveloped by a host cell membrane preventing direct contact with the cell cytoplasm and related organelles [2], [3]. Coculture of *H. pylori* with epithelial cell lines showed, in addition to bacterial uptake into the cells, the capacity of *H. pylori* to deliver toxins and other products into the cells, either by a type IV secretion system directly injecting CagA and peptidoglycan into the cytoplasm [4]–[6] or by the autotrasporter mechanism, as seen for VacA which then undergoes internalization through endocytosis [7], [8]. Endocytosis may also internalize outer membrane vesicles (OMVs) carrying several proteins (OMPs), lipopolysaccharides (LPS), and peptidoglycans (PGs) [9], [10]. Urease, a major component of *H. pylori* bodies, is known to be internalized by lamina propria cells [11] as well as luminal and intraepithelial granulocytes [12], while scarce evidence is available concerning epithelial cells.

Although it seems clear, at least from *in vitro* studies, that *H. pylori* products can enter epithelial cells, at present there is limited *in vivo* evidence concerning their internalization and intracellular fate in human gastric epithelium. In addition, it is not clear whether intracellular *H. pylori* may lose their enveloping host membrane, as it has been shown to occur for other bacteria [13], thus allowing free intracellular release of their virulence factors and direct contact with cytosolic components like NODs or the ubiquitin-proteasome system (UPS), known to be involved in intracellular bacterial recognition and management [6], [14].

Intracellular NOD receptors play an important role in *H. pylori* sensing by epithelial cells [6], [10] which respond to the bacterium or its virulence factors by releasing cytokines and chemokines which recruit and activate immune-inflammatory cells [15] or through enhanced production of HLA peptides, costimulatory molecules and cathepsins known to take part in antigen processing and presentation [16]–[18]. In addition, increased epithelial cell proliferation [19], altered apoptosis [20]–[22], and a variety of “cytotoxic changes” [1], [17] have been described, which may play a role in the genesis of the main pathologic sequelae of chronic *H. pylori* gastritis, such as peptic ulcer and cancer. However, several intracellular processes linking *H. pylori* virulence factors to the epithelial response in the infected human mucosa remain to be clarified, especially concerning type of cells and organelles or subcellular compartments involved.

During a recent investigation of human gastric mucosa, both *H. pylori* and its virulence factors were found to accumulate selectively into endosomal vesicles, phagolysosomes and the cytoplasm of dendritic cells, granulocytes, macrophages and mast cells, with or without associated cytotoxic changes [12]. Thus, thorough reinvestigation of *H. pylori*-infected human gastric epithelium seemed to be warranted so as to gain more evidence on possible cellular accumulation of bacterial products, their intracellular fate and pertinent host cell responses. The following questions were given special consideration: 1) Are bacterial products accumulated inside gastric epithelium, and in which cellular compartments? 2) Do “naked” (i.e., devoid of host enveloping membrane) *H. pylori* occur intracellularly so as to directly contact cytosolic host components? 3) Are bacterial products like VacA and outer membrane components, known to be taken up by endosomal vesicles of infected cells, released to the cytosol where sensors like NODs or UPS may sense them?

Here we demonstrate the existence of a novel intracellular structure we named PaCS (for Particle-rich Cytoplasmic Structures) where *H. pylori* products concentrate *in vivo* in colonized gastric epithelium as well as *in vitro* in human epithelial cell lines incubated with *H. pylori* products. PaCS, characterized by 13-nm-thick cylindrical particles we identified as proteasome complexes, is a distinctive cytoplasmic compartment where both NOD1, a selective *H. pylori* receptor, and UPS components are co-concentrated. This structure may have a role in bacterial recognition and handling, and may modulate the activity of toxins/virulence factors and induce pertinent immune responses, especially through immunoproteasome. The presence of PaCSs in gastric preneoplastic lesions, and their selective colocalization with putative oncogenic factors like CagA, SHP2 and members of the MAPK/ERK signaling system, may suggest this structure has a role in the regulation of cell growth and neoplastic transformation, also in view of its highly enriched UPS content and the mounting evidence for a role of UPS in cancer origin or progression.

## Results

### Identification of a discrete particle-rich cytoplasmic structure

**Figure 1:**
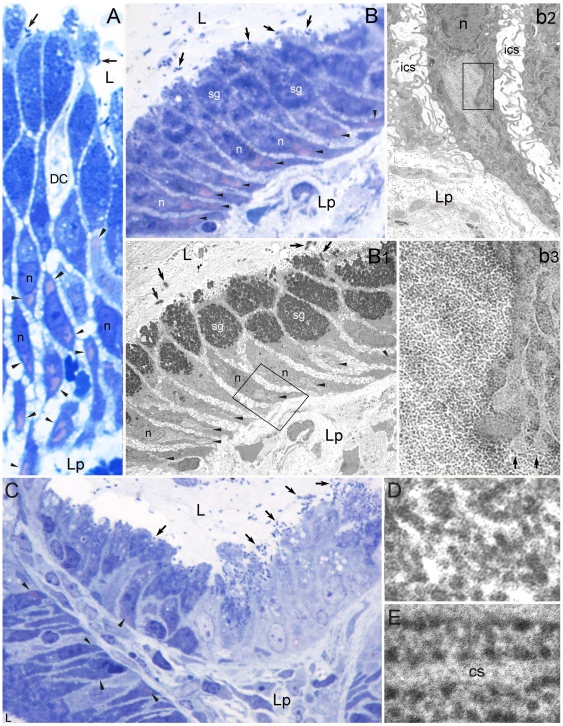
Identification of PaCS in *H. pylori*-colonized human gastric epithelium *in vivo*. (**A**, 1,000x) and (**B**, 1,000x) *H. pylori* (arrows) colonized superficial gastric epithelium stained with toluidine blue shows metachromatic pink areas (arrowheads), mainly infranuclear, surrounded by blue stained (ribosome-rich) cytoplasm. Note in **A** a dendritic cell (DC; see [ref. 12]) approaching luminal bacteria. Aldehyde-osmium fixed, 0.5-µm-thick resin sections. (**B1**, 1,000x) TEM of the same epithelium in a consecutive section to **B** shows identity of the pink structures with PaCSs. A PaCS is enlarged in **b2** (6,300x) and further in **b3** (31,500x) to show the characteristic particles (left side in **b3**), to be compared with ribosomes of surrounding RER (right side in **b3**). Note in **b3** direct contiguity of the PaCS with ribosomes and, in the lower right corner, with two RER cisternae (arrows), apparently without admixture of respective contents. (**C**, 500x) Toluidine blue stained resin section showing on the right a highly colonized, severely damaged epithelium with deeply irregular luminal border (due to cell bulging, desquamation and microerosion), vacuolation and loss of mucin granules in the apical-supranuclear cytoplasm, disappearance of ribosome-related basophilia in the basal cytoplasm and loss of cell polarity, to be compared with a relatively preserved epithelium in the lower left corner and a moderately damaged epithelium upper left. Note several metachromatic areas in lower left (arrowheads), a single fainty stained area upper left and no metachromatic areas in the severely damaged epithelium on the right. (**D**, 120,000x) High-resolution TEM of barrel-like, randomly oriented PaCS particles, 13 nm thick and 15 to 43 nm long, to show their regular punctate substructure. Compare with more dense, frequently angular ribosomes aligned along RER cisternae in **E** (120,000x). cs, cisterna; ics, intercellular space; L, gastric lumen; Lp, lamina propria; n, nucleous; sg, secretory granules.

**Figure 2:**
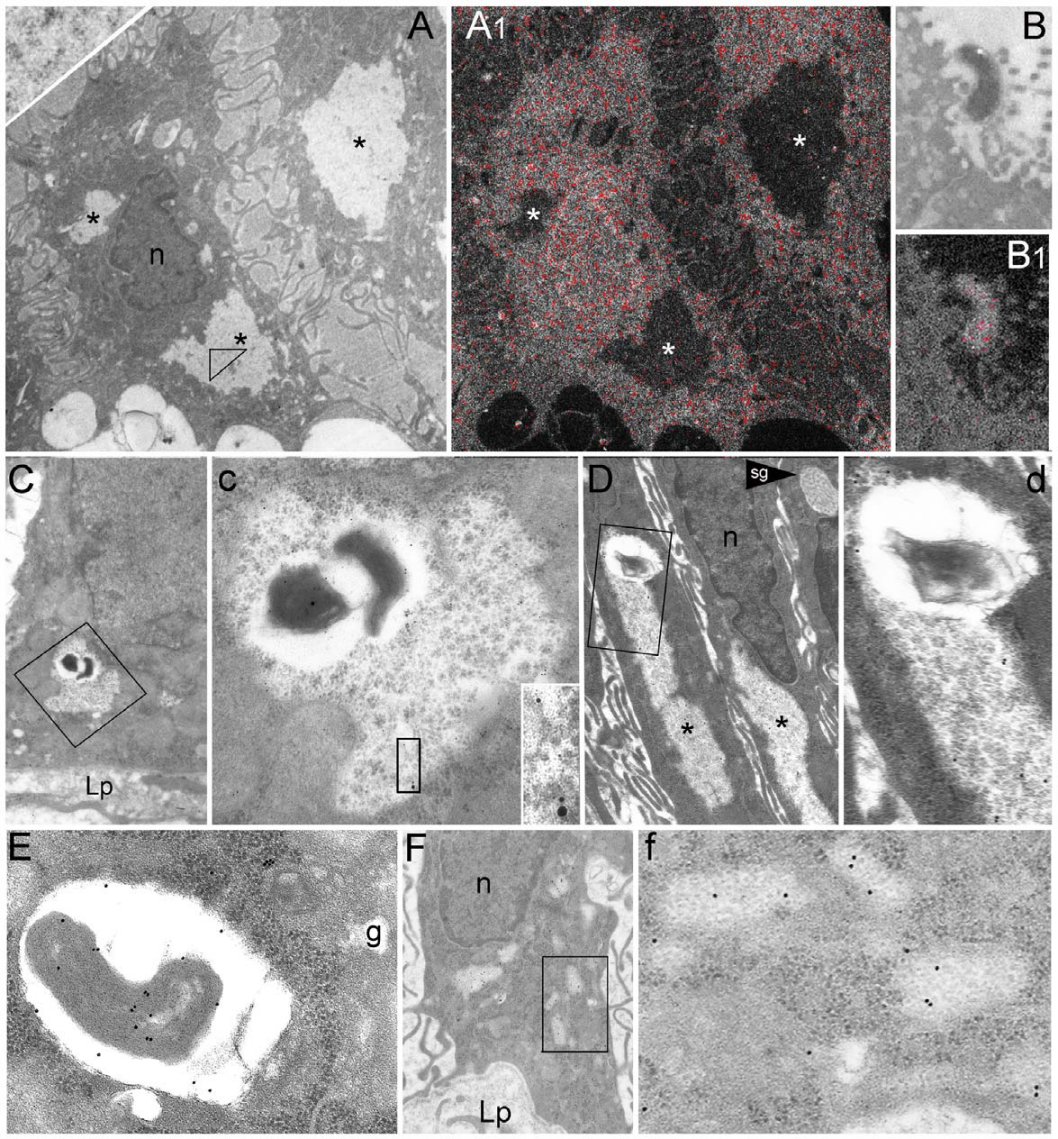
Characterization of PaCS in *H. pylori*-colonized human gastric epithelium *in vivo*. (**A**) and (**A1**) (both 4,000x) In two foveolar cells (***A***) three PaCSs (asterisks), one of which enlarged in the inset (upper left corner, 50,000x) to show their particles, lack phosphorus signal (red colour) when viewed under ESI analysis (**A1**). Note the intense signal of nucleoproteins in the nucleous, cytoplasmic ribosomes surrounding PaCSs and, in **B** and **B1** (both 8,000x), a luminal *H. pylori* adhering to epithelial cell microvilli. (**C**, 6,300x) and (**D**, 6,300x) *H. pylori* bodies inside PaCSs, two of which fairly-well preserved (**C**; enlarged in **c**, 16,000x), the other (**D**; enlarged in **d**, 16,000x) heavily degenerated. Note a peribacterial clear space, the immunogold reactivity of both bacteria and PaCS for *H. pylori* OMPs (5 nm gold) and VacA (10 nm gold) in **c** and its inset (44,000x), while the PaCS only, but not bacterial remnants, reacts for human 20S-β5i subunit of the immunoproteasome (**d**). (**E**, 25,000x) OMP-reactive intracellular *H. pylori* enclosed in a supranuclear vacuole of a foveolar cell. (**F**, 6,300x) Several small, thin PaCSs are sparse inside the RER at the base of an epithelial cell, enlarged in **f** (32,000x) to show their particles clearly less dense than ribosomes, as well as their selective immunogold reactivity for CagA. g, Golgi area; Lp, lamina propria; n, nucleous; sg, secretory granule.

Conventional paraffin sections of human gastric mucosa showed 19 patients with gastritis, among the 26 cases investigated, 15 of whom with *H. pylori* on the luminal side of their superficial-foveolar epithelium. Toluidine blu-stained semithin resin sections from the same cases allowed epithelial degenerative-cytotoxic changes to be identified more easily and revealed small pink-stained cytoplasmic areas inside the blue-stained infra/perinuclear cytoplasm of the foveolar cells (Figure 1A, B and C). Transmission electron microscopy (TEM) of consecutive sections identified such areas with well-defined, discrete cytoplasmic structures, situated in rough endoplasmic reticulum (RER)-rich cytoplasm and characterized by the accumulation of moderately osmiophilic barrel-like particles of about the same size as ribosomes. However, unlike ribosomes, the particles showed a distinctive punctate substructure due to minute spots forming parallel lines oriented orthogonally to the particle main axis (Figure 1B1, b2, b3, D, and E). Unlike ribosomes and other phosphonucleoprotein-containing structures, such particles showed no phosphorus signal when analyzed by electron spectroscopy imaging (ESI) (Figure 2A and A1, B and B1). From now on, we will refer to the Particle-rich Cytoplasmic Structures as PaCSs. Common cytoplasmic organelles like mitochondria, Golgi, lysosomes, endosomes, centrosomes or microtubules were generally excluded from PaCSs or pushed to their borders.

### *H. pylori* and their products inside PaCS

**Figure 3:**
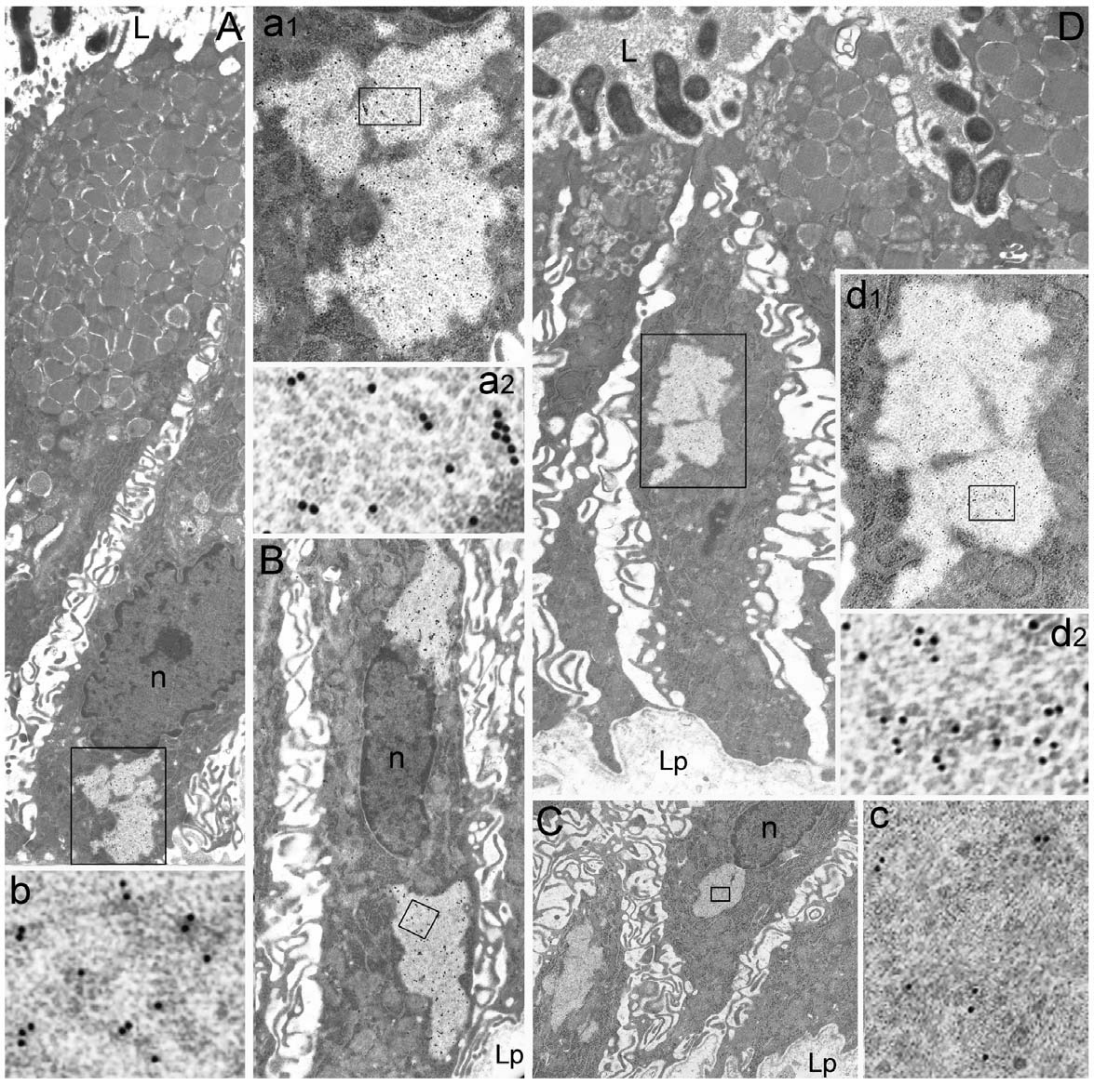
CagA, urease, VacA, and polyubiquitinated proteins accumulate in PaCS. Foveolar-superficial cells with luminal *H. pylori* localization, in the apparent absence of intracellular bacteria (**A**, 8,000x), show several PaCSs with selective immunoreactivity for: CagA (enlarged in **a1**, 12,000x, and in **a2**, 84,000x); urease (**B**, 8,000x), with PaCSs on both sides of the nucleous, one of which enlarged in **b** (84,000x); VacA (**C**, 4,500x; one enlarged in **c**, 60,000x); and polyubiquitinated proteins (**D**, 9,500x; enlarged in **d1**, 12,000x, and further in **d2**, 84,000x). Note in **A** and **D** remnants of cytoplasm inside PaCSs, suggesting their origin from enlargement and fusion of smaller structures like those in Figure 2F. L, gastric lumen; Lp, lamina propria; n, nucleous.

Sparse intraepithelial, intercellular or intracellular, bacterial bodies or their *H. pylori*-immunoreactive remnants were detected by TEM in most biopses examined, including specimens from 7 of 11 cases lacking bacteria on the luminal side and diagnosed as *H. pylori*-negative at light microscopy investigation of paraffin sections. Sometimes, *H. pylori* bodies or remnants were found inside PaCSs (Figure 2C and D), usually in cells also showing *H. pylori* outside PaCS, in membrane-enclosed vacuoles of the supranuclear cytoplasm (Figure 2E). Inside PaCS the bacteria, although frequently surrounded by a clear empty space, were devoid of host membrane envelopment, thus being allowed to interact with PaCS components (Figure 2c and d). The *H. pylori* nature of such bacterial bodies was confirmed by their immunogold reactivity for urease, VacA, and *H. pylori* OMPs (Figure 2c). In addition, PaCS itself, either with (Figure 2c) or without (Figures 2F and 3A, B and C) inner bacteria, also reacted selectively for bacterial products like VacA, CagA, urease or OMPs. Median immunogold particles counts inside at least 100 µm^2^ PaCS sections ranged from 3.41 (OMPs) to 3.68 (VacA), to 4.33 (urease) per µm^2^, with a ratio between PaCS and surrounding cytoplasm ranging from 8.5 (OMPs) or 8.6 (VacA) up to 27.1 (urease). CagA immunoreactivity of PaCS changed prominently in different biopsies, some of which being unreactive while others showing selective, though variable reactivity (from 1.05 up to 9.90 particles/µm^2^, and a PaCS/cytoplasm ratio ranging from 4.0 up to 12.8, partly depending on different antisera employed).

PaCSs apparently arise in the cytoplasm interposed between RER cisternae by focal, progressive concentration of their distinctive particles at sites where bacterial products accumulate (Figure 2F and f), with subsequent dislocation of surrounding cytoplasm and its organelles. Indeed, thin cytoplasmic remnants were sometimes found inside a PaCS, suggesting it originated from expansion and fusion of smaller foci (Figure 3A, a1, D, d1).

### PaCSs differ from VacA-induced vacuoles

**Figure 4:**
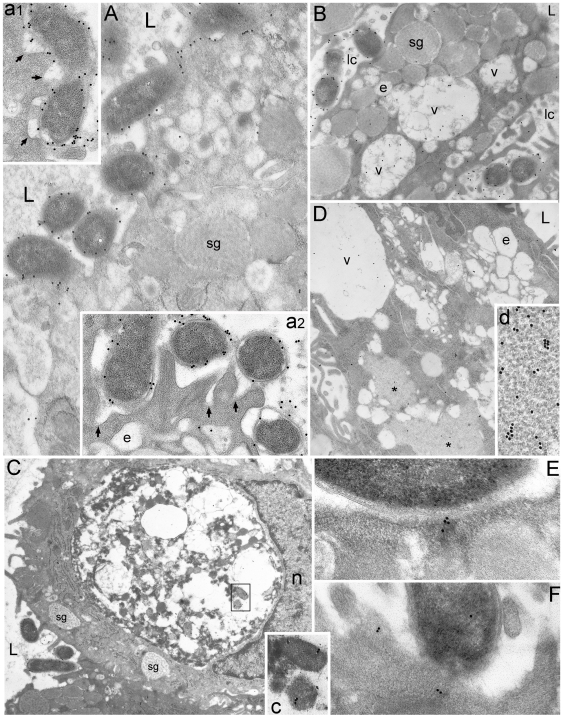
PaCSs differ from VacA-containing vacuoles and phagosomes. (**A**, 12,000x, 10 nm gold), (**a1**, 20,000x), and (**a2**, 20,000x) *H. pylori* organisms closely adhering to the luminal surface of colonized cells in superficial gastric epithelium. Note VacA immunoreactivity in bacterial periplasm, cellular early carriers under formation immediately below bacterial adhesion (arrows) and some apical endocytic-endosomal vesicles. (**B**, 7,000x) Endoluminal bleb of a colonized superficial cell showing immunoreactive VacA in adhering bacteria and some endosomal vesicles fusing each other to form VacA-storing larger vacuoles with inner remnants of original endosomal membranes. (**C**, 10,000x) Colonized cell showing a large supranuclear phagosome with abundant cytoplasmic remnants and debris and VacA immunoreactivity, enlarged in **c** (40,000x). (**D**, 7,000x) HeLa cells incubated for 24 h with *H. pylori* BCF. Note inside the cell VacA immunoreactivity of some endosomal vesicles, a large vacuole as well as two PaCSs (asterisks) with distinctive particles (**d**, 60,000x). (**E**) and (**F**) (both 55,000x) Small CagA deposits in the subluminal cytoplasm just below two adhering *H. pylori*. L, gastric lumen; foveolar cell type; e, endosomal vesicle; lc, luminal cleft; n, nucleous; sg, secretory granules; v, vacuole.

In the majority of cases, *H. pylori* products stored in PaCS are likely to take origin, as well as from the relatively few intracellular bacteria, from more abundant extracellular bacteria colonizing gastric epithelial cells on their luminal side (Figure 3A and D). Indeed, many membrane invaginations (Figure 4A, a1 and a2), often carrying VacA labelling, were found at sites of bacterial adhesion. They most likely correspond to the “early VacA carriers” seen *in vitro* to enter cell cytoplasm of VacA-incubated HeLa cells and form tubular early endosomes [8]. Indeed, many tubular vesicles, sometimes VacA-storing, were seen in the apical cytoplasm of colonized superficial-foveolar cells, together with distended round vesicles probably corresponding to late endosomes (Figure 4A and B). We also observed reciprocal fusion of the latter to form larger VacA-storing vacuoles, still showing remnants of the original endosome membrane inside, especially in endoluminally bulging cells bordered by intercellular luminal clefts (Figure 4B). In addition, autophagic vacuoles storing various cytoplasmic remnants and dense bodies, as well as VacA immunoreactivity were frequently found in the supranuclear cytoplasm (Figure 4C and c).

*In vitro* experiments on HeLa cells incubated for 24 h with *H. pylori* broth culture filtrate (BCF), besides confirming previous observations on VacA (and OMPs) uptake into endosomal vesicles and vacuoles [7], [9], clearly identified typical PaCSs showing VacA immunoreactivity (Figure 4D and d), thus proving their independence from VacA-storing vacuoles at ultrastructural level, despite sharing VacA content. It should be noted that, at variance with *in vitro* non-polarized cells, where PaCSs and VacA-storing vacuoles were usually admixed in the same cytoplasmic areas (Figure 4D), in superficial-foveolar cells of gastric biopsies they were topographically separated, with PaCSs mainly located in the deep infra/perinuclear, ribosome-rich cytoplasm and VacA-storing endosomes, vacuoles or phagosomes in the ribosome-poor, apical/supranuclear cytoplasm.

Concerning CagA, small, discrete immunogold deposits were found in the foveolar cell cytoplasm immediately below some adhering bacteria (Figure 4E and F), a finding which may be compatible with a non-endocytotic way of entry.

### NOD1 receptor in PaCS

**Figure 5:**
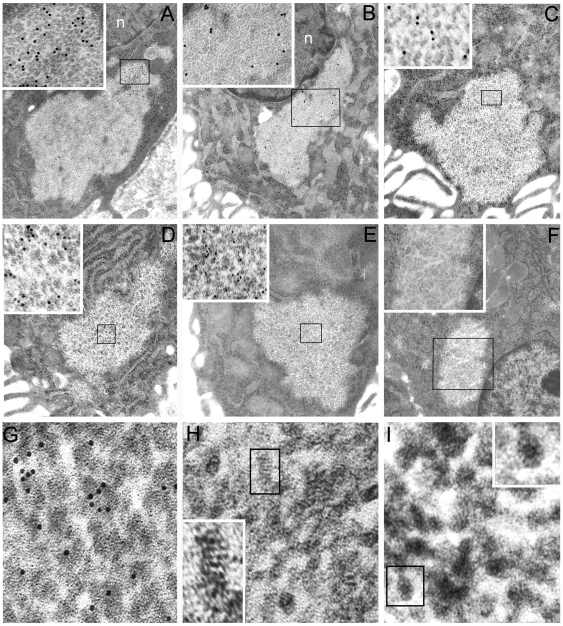
Proteasome is the particle component of PaCS which also contains NOD1. Selective PaCS reactivity for NOD1 (**A**, 12,500x; boxed part enlarged in its inset, 60,000x); ubiquitin-activating enzyme E1 (**B**, 10,000x; enlarged in its inset, 32,000x); 20S proteasome (**C**, 12,500x; enlarged in its inset, 60,000x); 20S-β5i subunit of immunoproteasome (**D**, 12,500x; enlarged in its inset, 60,000x); and 19S proteasome (**E**, 12,500x; enlarged in its inset, 60,000x). Note in **F** (10,000x) and its inset (32,000x) lack of immunogold particles in a PaCS and surrounding cytoplasm of a control section from the same resin block (as in **A**, **B**, and **C**) incubated with gold-labelled non-immune globulins. In **G** (125,000x), **H** (340,000x) and **I** (340,000x) high-resolution TEM of the 13-nm-thick PaCS particles, selectively immunoreactive for 19S proteasome (**G**), shows their inner punctate substructure with minute electrondense spots aligned perpendicularly to particle long axis. On a side view like that further enlarged in the inset of **H** (600,000x), some particles closely resemble the four parallel rings of a proteasome 20S core capped at both estremities, while on a top view like that in the inset of **I** (600,000x) they may reproduce the proteasome seven-fold star-like symmetry. n, nucleous.

As NOD1 has been identified as a major intracellular *H. pylori* sensor of epithelial cells [6], we tested NOD1 antibodies on *H. pylori*-colonized human superficial-foveolar cells of gastric biopsies. As shown in Figure 5A, NOD1 immunoreactivity was found to be selectively concentrated in PaCSs. Interestingly, the toluidine blue metachromatic basophilia found in PaCS (Figure 1A and B) is a known property of anionic glycoconjugates [23], to which proteoglycans, the specific ligands of NOD1 [24], belong. As to the nature of the compounds accounting for PaCS metachromatic basophilia, we found that, when the pH of the toluidine blue solution was progressively lowered from 8.0 (i.e., the usual value of the standard borax solution used for staining semithin resin sections) down to 3.0, metachromatic basophilia persisted down to pH 4.2, weakened at pH 3.9 and completely disappeared at pH 3.6. This behavior should rule out strong anions like sulphates or phosphates (the latter also being ruled out by ESI analysis (Figure 2A1)) while favouring the involvement of relatively weak anions such as carboxylated glycoconjugates like bacterial proteoglycans [24].

### UPS components in PaCS

**Figure 6:**
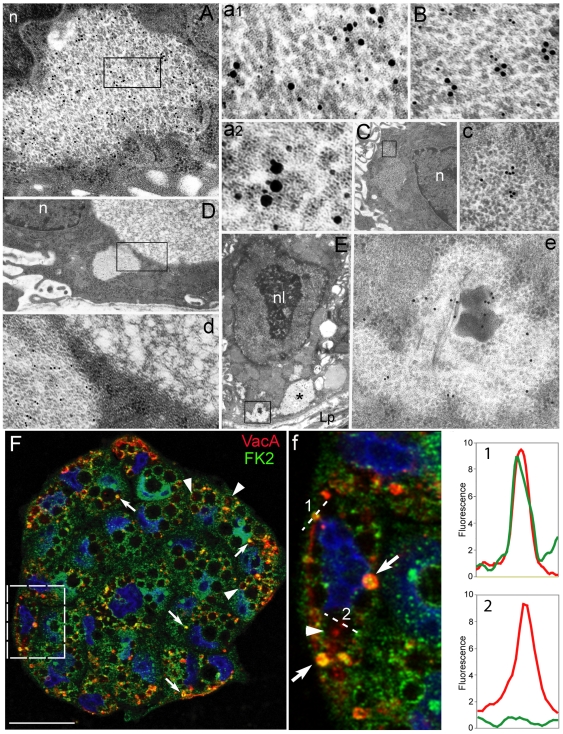
Colocalization in PaCS of bacterial products, UPS and oncogenic/signaling molecules *in vivo* and *in vitro*. (**A**, 20,000x; enlarged in **a1**, 95,000x, and **a2**, 120,000x) Colocalization in PaCSs of CagA (15 nm gold particles) with polyubiquitinated proteins (10 nm gold particles) and 20S-β5i subunit of immunoproteasome (5 nm gold particles). Note in **a1** and **a2** close reciprocal contacts of some 20-nm gold particles with 10- or 15-nm ones; also note in *a2* the linear-punctate substructure of PaCS particles. (**B**, 80,000x) In another section through the same PaCS as of **A**, colocalization of CagA (15 nm gold) and SHP2 protein (10 nm gold) is obtained. (**C**, 5,000x; enlarged in **c**, 35,000x) Another PaCS showing selective ERK reactivity. (**D**, 10,000x; enlarged in **d**, 45,000x) In addition to a typical particulate PaCS (left part of **d**) immunoreactive for FK1 antibodies (recognizing polyubiquitinated proteins), another, FK1-unreactive non-particulate cytoplasmic structure (upper right of **D** and **d**) with a filamentous-honeycomb meshwork. (**E**, 6,300x) Dysplastic cell with prominent nucleolus showing several PaCSs (one labelled with asterisk) in its basal cytoplasm. A PaCS containing bacterial remnants is enlarged in **e** (20,000x) to show *H. pylori* (OMPs) immunoreactivity of both bacterial body and PaCS itself. n, nucleous; nl, nucleolus. (**F**) Confocal microscopy of HeLa cells (nuclei in blue) incubated for 24 h with *H. pylori* BCF shows the presence in the cytoplasm of yellow spots (arrows; see also enlargement in **f**) representing the colocalization of ubiquitinated proteins (FK2; green) with VacA (red). Note that vacuole-associated VacA (arrowheads) does not colocalize with FK2. Colocalization charts (graphs 1 and 2) show the intensity profile for each fluorescence taken along the dotted lines. Pictures are from one single confocal section. Bar: 25 µm.

In addition to bacterial products and NOD1, PaCSs were found to show consistent and selective immunogold reactivity for polyubiquitinated proteins (Figure 3D, d1 and d2), ubiquitin-activating ligases E1A/B (Figure 5B) and proteasomal components like 19S, 20S and 20S-β5i subunits, usually in close topographic connection with PaCS inner particles (Figures 2D, and 5C, D, E and G). Therefore, we reconsidered the high-resolution ultrastructure of these particles in comparison with that reported by *in vitro* X-ray analysis of the proteasome complex [25]–[27]. In thin, appropriately oriented and sufficiently contrasted sections from both gastric biopsies and epithelial cell lines, PaCS particles showed a barrel-like form measuring around 13 nm in thickness and from 15 to 43 nm in length (Figures 1D, 5G, H and I, and 6a2). These measures fit in well with those reported *in vitro*, for the 20S proteasome complex or the 26S “capped” proteasome, respectively, where the 20S core particle is extended at one or both extremities by the addition of the 19S or the PA28 “cap” [25]–[27]. The four-ring substructure of the 20S core proteasome was also recognized in some side views of the PaCS particle, with (Figure 5H and its inset) or without addition of cap structures at its extremities. In addition, “top” views of favorably oriented particles, so as to provide a cross section of the barrel (Figure 5I and its inset), also showed the characteristic seven-star structural pattern reported for isolated proteasome complex [25].

Control immunogold tests with rabbit or mouse immunoglobulins gave negative results in PaCSs (Figure 5F), as did antibodies directed against ribosomal proteins, cathepsin D, S-100 protein or insulin and glucagon hormones. Selective PaCS reactivity was also found for some cytosolic proteins known to interact with CagA, as SHP2 tyrosine phosphatase (Figure 6B) which forms complexes *in vitro* and *in vivo* with CagA, or ERK kinases (Figure 6C) involved in a MAP kinase signaling pathway activated by CagA [28]. Tests on consecutive ultramicrotomic sections through the same PaCS or on the same section using multiple antibodies linked to gold particles of different size confirmed colocalization of each bacterial protein with NOD1, polyubiquitinated proteins and proteasomal components, sometimes with gold particles tightly adherent to each other so as to suggest possible interaction of respectively linked molecules (Figure 6A, a1 and a2).

### Non-particulate, UPS-negative structures

In addition to PaCS, other types of cytoplasmic structures were observed by TEM, less commonly in gastric epithelium and more frequently in epithelial cell lines. They showed a loose filamentous meshwork often forming an irregular honeycomb-like scaffold, or with foci of more compact fibrillar structure, in the absence of 13-nm-thick particles (Figure 6D and d). These particle-free, fibrillar to honeycomb-like structures were sometimes found to coexist with PaCS, adjacent to each other inside the same cytoplasmic area, occasionally with patterns suggesting that they may originate from PaCS by dissolution of the 13-nm particles with retention of the interparticle scaffold.

### PaCS distribution

Both PaCS under TEM and metachromatic areas of toluidine blue-stained resin sections under light microscopy were mainly found in superficial-foveolar cells of *H. pylori*-infected gastric mucosa, with progressively increasing number and size from the renewal zone to the surface epithelium. Less numerous or smaller PaCSs were seen in superficial-foveolar cells devoid of luminal bacteria, though showing sparse intracellular *H. pylori* and moderate mononuclear cell inflammation in the underlying lamina propria.

PaCSs were not observed in acidopeptic, pyloric or cardial gland cells, nor in complete, small intestine-type (type I) metaplastic epithelium or lamina propria cells of inflamed gastric mucosa. In three cases, we found areas of colonic-type (type III) intestinal metaplasia whose sparcely granulated goblet cells showed intracellular *H. pylori*, especially in the supranuclear cytoplasm [2], as well as typical PaCSs in the infranuclear cytoplasm. PaCSs were also found in dysplastic foci observed in two such cases (Figure 6E and e).

In *H. pylori*-positive mucosa, PaCSs were significantly less represented in epithelial cells showing cytotoxic changes like increased cell desquamation with minute erosions, severe irregularity of the luminal surface alternating cell bulging with intercellular luminal clefts, altered cell polarity, focal micropapillary hyperplasia of mucin-poor elongated cells, cytoplasmic vacuoles and autophagosomes (Figures 1C and 4A, B and C), compared with well-polarized cells lacking signs of severe epithelial damage (Figures 1A, B, B1 and 3A) and often coexisting in the same case or even in the same mucosal specimen (Figure 1C).

### Confocal microscopy of BCF-treated epithelial cells in culture

**Figure 7:**
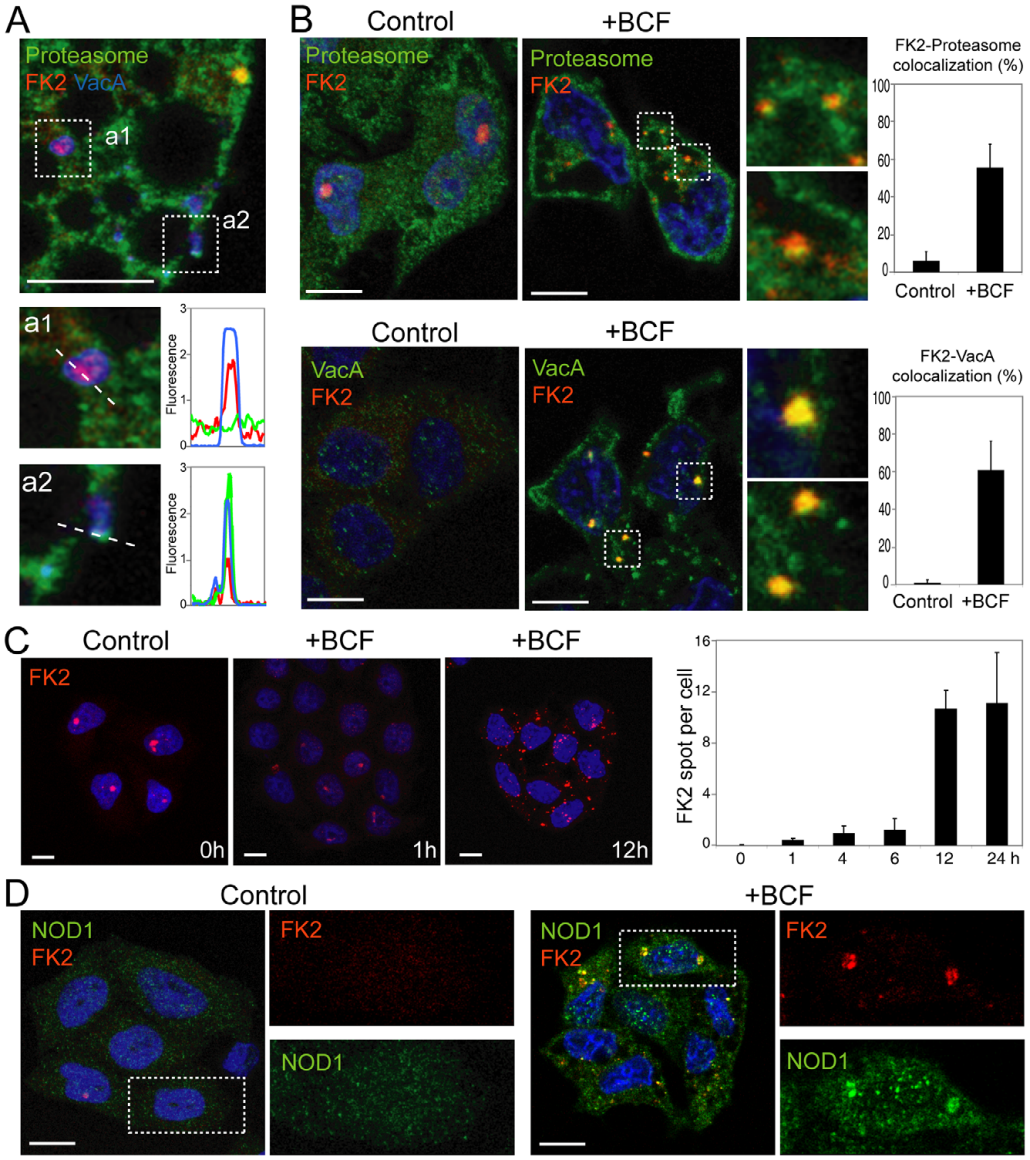
*H. pylori* products/virulence factors induce PaCS formation *in vitro*. (**A**) Confocal microscopy of HeLa cells incubated for 24 h with Cy5-labeled purified VacA for the simultaneous detection of proteasome (green), ubiquitinated proteins (FK2; red), and VacA (blue). The enlargements show in **a1** colocalization (purple) between ubiquitinated proteins and VacA, and in **a2** colocalization (light blue/white) of proteasome, ubiquitinated proteins, and VacA. The graphs on the right represent the respective colocalization charts showing the intensity profile for each of the three fluorescences taken along the dotted lines. Pictures are from one single confocal section. Bar: 10 µm. (**B**) Confocal microscopy of HeLa cells (nuclei in blue) incubated for 24 h without (control) or with *H. pylori* BCF shows the presence in the cytoplasm of BCF-treated cells of spots with the yellow component representing the colocalization of ubiquitinated proteins (FK2; red) with proteasome (green) (top line) or of ubiquitinated proteins (red) with VacA (green) (bottom line), respectively. These cytoplasmic colocalizations in either control or treated cells are quantitatively analyzed in the histograms (right) showing the percentage of FK2-positive spots colocalizing with proteasome or VacA, respectively. Pictures are from one single confocal section. Bar: 10 µm. (**C**) Time-course of HeLa cells (nuclei in blue) incubated with *H. pylori* BCF shows a time-dependent increase in the cytoplasmic spots of ubiquitinated proteins (FK2; red). The histogram (right) shows the number (means ± SEM) of FK2-positive cytoplasmic spots per cell. Bar: 10 µm. (**D**) Confocal microscopy of HeLa cells (nuclei in blue) incubated for 24 h without (control) or with *H. pylori* BCF shows the presence in the cytoplasm of BCF-treated cells of yellow spots representing the colocalization of ubiquitinated proteins (FK2; red) with NOD1 (green). Each individual labeling is shown in the enlarged areas of the boxed regions. Pictures are from one single confocal section. Bar: 10 µm.

Parallel confocal microscopy tests on BCF-treated HeLa or AGS cells confirmed ultrastructural findings by showing VacA in PaCSs, characterized by their concentration of ubiquitinated (i.e., FK2-reactive) proteins (Figure 6F). Indeed, in agreement with previous findings [7], [22], a large amount of VacA was associated to cytoplasmic vacuoles which, on the contrary, lacked ubiquitinated proteins (Figure 6F). PaCS identification at confocal microscopy level was further confirmed by additional colocalization of proteasome (Figure 7A), which, like VacA colocalization, was essentially due to BCF treatment (Figure 7B). Interestingly, after 24 h incubation with *H. pylori* BCF, HeLa cells still showed a proteasome-negative 45% fraction of FK2-reactive spots (Figure 7B). This fact, which is paralleled by a similar behavior of VacA/FK2 colocalization, may be explained by the time-dependence development of both FK2- (Figure 7C) and proteasome-reactive (not shown) spots. It seems likely that the whole process of PaCS formation is time-dependent, with final recruitment of the proteasome to the aggregate of bacterial products and ubiquitinated proteins. The role of *H. pylori* and its products in PaCS formation is further stressed by the enhanced colocalization between ubiquitinated proteins and proteasome in BCF-treated cells compared to untreated control cells (Figure 7B) and by the observation that colocalization between ubiquitinated proteins and NOD1 receptor was present only in BCF-treated cells (Figure 7D).

## Discussion

In this study a novel cytoplasmic structure is described in *H. pylori*-colonized human gastric epithelium which is essentially characterized by accumulation of regularly spaced, 13-nm-thick, elongated particles inside a relatively clear, amorphous to filamentous background, to form a distinct cytoplasmic area surrounded by RER. Such a particle-rich cytoplasmic structure (PaCS) showed selective reactivity for a) bacterial products like CagA, VacA, urease and OMPs, sometimes coupled with *H. pylori* bodies or remnants, b) NOD1 receptor for bacterial peptidoglycans, c) E1 ubiquitin-activating enzyme and polyubiquitinated proteins, and d) several proteasome components, including the 20S core subunits, 19S-S2 subunit, and 20S-β5i subunit (also known as LMP7, characterizing the INFγ-stimulated immunoproteasome). PaCSs apparently arise in the juxtaribosomal cytoplasm interposed between RER cisternae, at sites of bacterial products accumulation, and progressively grow by accumulation of newly formed particles, while pushing aside cytoplasmic organelles.

In thin sections analysed at high resolution, some of the PaCS particles showed ultrastructural resemblance to the structure reported *in vitro* by X-ray analysis of the proteasome complex [25]–[27], with which they are likely to be identified, as also suggested by their direct immunoreactivity for proteasomal components. Previous immunogold studies documenting a close topographic correlation between proteasome immunoreactivity and RER [29] may also support this conclusion, given present separation of phosphorous-positive, proteasome-negative, RER-associated ribosomes from the phosphorous-negative, proteasome-immunoreactive 13-nm particles collected inside PaCS.

PaCSs were more prominent in the basal cytoplasm of superficial-foveolar cells showing *H. pylori* colonization on their luminal side. Vesicular endosomes, often storing VacA, were seen to accumulate in the apical cytoplasm of colonized epithelium and to fuse each other to form VacA-storing vacuoles, as already shown *in vitro* [7], [30] and *in vivo* [9], [17]. Both electron and confocal microscopy of human gastric biopsies or cultured epithelial cells clearly separated PaCS from VacA-storing vacuoles and phagosomes, despite their common toxin content. In addition, minute, free CagA deposits were seen in the cytoplasm just underlying *H. pylori* adhesion sites. How both cytosolic (CagA) and endocytosed (VacA and OMPs) bacterial products reach deeply situated PaCS (an essentially cytosolic compartment) after entering the apical part of the cell, remains to be clarified.

PaCSs were also observed in biopses from cases showing no *H. pylori* at routine light microscopy investigation of paraffin sections. However, in many such cases TEM detected intracellular *H. pylori* bodies or their remnants, more frequently in the supranuclear cytoplasm and enveloped by a host membrane. Occasionally, they were also found in RER-rich basal cytoplasm, usually devoid of host enveloping membrane while directly contacting UPS-reactive 13-nm particles to form a PaCS. Such a pattern supports the involvement of *H. pylori* and/or its products in the genesis of at least a part of the PaCSs *in vivo*, while fitting with PaCS neogenesis and expansion in cultured cell lines incubated with *H. pylori* BCF.

The colocalization of *H. pylori* products with NOD1 inside the PaCS is especially interesting in view of the demonstration that this receptor has a crucial role in sensing *H. pylori* PG to elicit NF-kB activation and chemokine-dependent proinflammatory response [6]. We also colocalized with NOD1 in PaCS both OMPs, known to be linked to PGs and LPS inside *H. pylori* outer membrane and related vesicles [9], [10], and a moderately acidic polyanion conferring selective metachromatic basophilia of the type displayed by carboxylated glycoconjugates like PGs [23], [24].

Of major interest is also the colocalization of bacterial products with ubiquitin-activating enzyme E1, polyubiquitinated proteins and proteasome components since interactions between bacterial toxins/virulence factors and the UPS of infected cells are well-known to occur [14], [31], [32] resulting in a modulated host cell response to bacteria. As shown for *Salmonella typhimurium*, when intracellular bacteria escape from their membrane-enclosed invasion vacuole to directly contact host cell cytosol, the ubiquitin system may also have a role in recognizing and degrading them through selective proteasome recruitment into peribacterial cytosol [13]. These findings are reminiscent of the *H. pylori* bodies showing various degenerative changes that we found lying free inside some PaCSs of human gastric mucosa, in direct contact with UPS components. Direct interaction of *H. pylori* or its products and UPS may underscore an attempt by colonized epithelial cells to dispose (or modulate activity) of bacteria and their toxins/virulence factors. Bacterial products/UPS colocalization was also observed inside minute collections of proteasome-like particles first appearing in the cytoplasm adjacent to RER-bound or free polyribosomes, a likely site of origin of PaCSs. This finding may suggest that UPS and the PaCS particles are newly synthesized at a ribosomal level (or preferentially redistributed there) in response to bacterial products accumulation. Indeed, immunoproteasome has been shown to increase substantially at a RER/ribosomal level after microbial incubation [33], [34], resulting in proteasome-mediated bacterial antigen release associated with stimulation of class I MHC peptide neosynthesis and activation of specific immune responses [35].

In bioptic specimens, we found UPS-positive PaCSs a) in superficial-foveolar gastric epithelium, especially when showing heavy luminal *H. pylori* colonization in the absence of cytotoxic changes, b) in mucin-poor goblet cells of colonic-type (type III) intestinal metaplasia, with or without dysplastic changes, showing intraepithelial (but not luminal) *H. pylori*, and c) albeit less frequently, in superficial-foveolar cells lacking luminal bacteria while showing sparse intracytoplasmic *H. pylori*. In control cultured cells, UPS-positive PaCSs were observed only occasionally and in the absence of any *H. pylori* product-related reactivity. Incubation of the same cell lines with *H. pylori* BCF induced formation, enlargement and quantitative enhancement of structures positive for *H. pylori* toxins/virulence factors, NOD1, ubiquitinated proteins and proteasome, which closely reproduced cytochemical and ultrastructural patterns of corresponding *in vivo* structures, thus confirming a significant role of *H. pylori* in their development.

Other structures, unreactive for proteasome components, bacterial products or NOD1 and essentially formed by a meshwork of filamentous proteins resembling that reported by Simonsen *et al.* [36] and Kaganovitch *et al.* [37] in autophagy-related structures, were found occasionally in gastric foveolar epithelium and, more frequently, in cultured cell lines. A variety of discrete cytoplasmic structures potentially related to PaCS or filamentous structures has indeed been reported in epithelial and non-epithelial, cell lines, especially under conditions (including bacterial LPS treatment) altering the quality control of endogenous or exogenous, natural or mutated, misfolded proteins [38]–[42]. Among such previously described structures, only the ALIS (aggresome-like induced structure) caused by intracellular *Legionella pneumophila* in macrophages and dendritic cells [42] was induced by incubation with a bacterium. In that case, ALIS showed colocalization of ubiquitinated proteins and proteasome, thus resembling immunocytochemically the *H. pylori*-induced PaCSs, although no information on its high-resolution ultrastructure was given. The RER-associated DALIS (dendritic cell aggresome-like induced structure) [38] or ALIS [40], [41] induced *in vitro* by bacterial LPS in dendritic cells, macrophages and epithelial cells, though rich in ubiquitinated proteins, lacked proteasome reactivity. It remains to be ascertained, through further ultrastructural studies, whether PaCSs are akin to cytochemically similar structures arising in conditions of increased misfolded proteins production such as the UPS-positive JUNQ (juxtanuclear quality control compartment) [37], as infection may cause neosynthesis of misfolded proteins [43], [44].

Much work remains to be done, especially at molecular level, to understand the biologic and pathologic role of PaCS. Although *H. pylori* infection appears to induce or enhance its formation, PaCS is not part of the severe cytotoxic changes arising in the epithelium at sites of more heavy colonization by tightly adhering bacteria. These more “acute” inflammatory and cytopathic effects affecting epithelium integrity and barrier function seem likely to have a direct role in the genesis of ulcerative disease [17], [45]. On the other hand, epidemiologic, histopathologic and molecular studies suggest a relevant role of chronic *H. pylori* infection, with special reference to CagA-positive strains, in gastric carcinogenesis [2], [46]–[50] through a long-standing sequence of inflammatory, atrophic, metaplastic and dysplastic changes leading to intestinal-type cancer [48], [51]. Recent molecular studies have clarified in part the role of CagA in this process by showing complex formation with the oncogenic protein SHP2 and activation of the ERK kinases pathway so as to elicit proliferative, morphogenetic and antiapoptotic effects of potential neoplastic relevance [19], [21], [28]. Our finding of selective colocalization of CagA, SHP2 and ERK kinases inside some PaCS may suggest this structure has a role in the regulation of cell growth and neoplastic transformation, also in view of its highly enriched UPS content and the mounting evidence for a role of UPS in cancer origin or progression [52]. PaCS detection, together with bacteria and their virulence factors, inside gastric precancerous lesions is in keeping with this suggestion and, more in general, with the hypothesis of a *H. pylori*/CagA-mediated carcinogenesis.

In conclusion, it seems clear that PaCS, the novel intracellular structure where *H. pylori* products concentrate *in vivo* in colonized gastric epithelium as well as *in vitro* in human epithelial cell lines incubated with *H. pylori* products/virulence factors, is a distinctive cytoplasmic compartment where both NOD1, a selective *H. pylori* receptor, and UPS components are co-concentrated. This structure may have a role in bacterial recognition and handling, and may modulate the activity of toxins/virulence factors and induce pertinent immune responses, especially through immunoproteasome. The presence of PaCSs in gastric preneoplastic lesions, and their selective colocalization with putative oncogenic factors like CagA, SHP2 and members of the MAPK/ERK signaling system, add special interest to the investigation of the biologic role and pathologic potential of PaCS.

## Materials and Methods

Biopsy samples, retrieved from our archival histology collection, were taken in the period 1981–95 from the gastric antrum and corpus of 26 subjects (15 males and 11 females, aged between 26 and 79 years) undergoing routine endoscopic and histologic examination for dyspepsia as requested by the physician in charge of the patient and with the written consent of the patient. One of the specimens from both the antrum and the corpus was processed for TEM and the pertinent semithin resin section used for diagnostic purposes together with those of routine histologic material. No specimen specifically and/or exclusively devoted to the present study was taken. The study has been approved by the Ethics Committee of Fondazione IRCCS Policlinico San Matteo (Pavia, Italy) as a reinvestigation of archival material along the same line (i.e., diagnosis of *H. pylori*-dependent gastritis) as for the original consensus.

The samples were fixed in 4% formaldehyde and embedded in paraffin for histologic investigation, or fixed for 4 hours with 2% formaldehyde and 2.5% glutaraldehyde in 0.1 M phosphate buffer (pH 7.3), followed by 1% osmium tetroxide for 1 hour, embedded in Epon-Araldite resin and processed for TEM. Paraffin sections were stained with hematoxylin-eosin, Giemsa or *H. pylori* immunoperoxidase [2], [17]. Semithin (0.5 µm) resin sections were stained with toluidine blue, while ultrathin sections were stained with uranyl-lead or the immunogold procedure [2], [9], using antibodies against: a) *H. pylori* OMPs, urease, CagA and VacA, b) NOD1 receptor, c) E1A/B ligases, polyubiquitinated or mono/polyubiquitinated proteins, 20S proteasome core subunits, 19S proteasome S2 subunit, and 20S proteasome β5i subunit, d) SHP2 tyrosine phosphatase, ERK 1/2 kinases, ribosomal protein S16 and other proteins (detailed in Table S1). Anti-rabbit or anti-mouse IgG labeled with 5, 10, 15 or 20 nm gold particles (British Bio Cell, Cardiff, UK) were then used. Tests to evaluate the specificity of immunogold labeling were carried out using antibodies absorbed with excess antigen and omitting or substituting the specific antibodies in the first layer of the immunogold procedure. Positive and negative controls were obtained by parallel investigation of *H. pylori* cultures, epithelial cell cultures, and gastric mucosa specimens as in previous studies [2], [9].

ESI analyses were performed by using a LEO 912AB electron microscope as described by Pezzati *et al.* [53]. Briefly, the net phosphorus distribution was obtained by computer processing of images collected at different energy loss values according to the three-window method. The final phosphorus map (coded in pseudocolors) was then superimposed on the ultrastructural organization of the same field obtained at 250 eV (i.e., at an energy loss where most of the elements contribute to the image).

*H. pylori* BCF was produced as previously described [7] using the well-characterized urease^+^/CagA^+^/VacA^+^ wild-type *H. pylori* strain 60190 (ATCC 49503). Briefly, bacteria were grown in Brucella broth (BD Diagnostics, Sparks, MD) supplemented with 1% Vitox (Oxoid, Basingstoke, UK) and 5% fetal bovine serum (FBS; Gibco, Grand Island, NY) for 24–36 h at 37°C under microaerobic conditions and continuous shaking. Bacteria were then removed by centrifugation and the supernatant sterilized by passage through a 0.22 µm cellulose acetate filter. Cultured cells were incubated with *H. pylori* BCF diluted 1∶3 in culture medium.

VacA (with a s1a/m1 *vacA* genotype) was purified from BCF of *H. pylori* 60190 strain, grown in Brucella broth containing 0.2% β-cyclodextrins (Sigma, St Louis, MO) instead of FBS, by ammonium sulphate precipitation and gel filtration chromatography in accordance with Cover *et al.* [54]. Purified VacA was then labeled with Cy5 dye, stored in melting ice and, immediately before use, alkali activated by drop-wise addition of 0.4 N NaOH [8]. Cultured cells were incubated with 2 µg/ml activated Cy5-VacA.

Human epithelial cell lines HeLa (ATCC CCL-2; from cervix adenocarcinoma) and AGS (ATCC CRL-1739; from gastric adenocarcinoma) were grown in DMEM supplemented with 10% FBS and 200 mM glutamine at 37°C in a humidified atmosphere of 5% CO_2_ in air. After washing, subconfluent cell monolayers were incubated at 37°C for 24 h under the different experimental conditions. Cells were then either fixed and processed for TEM as described above or fixed with 4% paraformaldehyde, permeabilized with 0.1% saponin, and processed for immunofluorescence as previously described [8], [22] using Alexa 488-labeled anti-mouse IgG (Molecular Probes, Eugene, OR) or Texas Red- or Cy5-labeled anti-rabbit IgG (Jackson Immunoresearch, West Grove, PE) as secondary antibodies. Nuclear counterstaining was made with Hoechst 33258. TCS SP2 confocal laser scanning microscope (Leica, Heidelberg, Germany) equipped with 63x oil-immersion objective was used. Ubiquitinated protein-positive (i.e., FK2-reactive) cytoplasmic spots per cell and their colocalization with VacA or proteasome were quantified using the ImageJ software (NIH, Bethesda, MD).

## Supporting Information

Table S1 Characteristics of antibodies used for TEM and confocal microscopy.
(0.02 MB PDF)
