# Supplementary material for: In Vivo Accumulation of Helicobacter pylori Products, NOD1, Ubiquitinated Proteins and Proteasome in a Novel Cytoplasmic Structure
Source: PLoS One. 2010 Mar 16;5(3):e9716. doi: 10.1371/journal.pone.0009716 (PMC2838800; doi:10.1371/journal.pone.0009716)
Supplement: Table S1 — Characteristics of antibodies used for TEM and confocal microscopy. (0.02 MB PDF) [file pone.0009716.s001.pdf]

Table S1. Characteristics of antibodies used for TEM and confocal microscopy

**Antigens**	**Antibodies**	**Source**	**Code**	**Working dilution**
H. pylori OMPs	RP, 1.5 mg protein/mL	*A*	V4074	1:200
H. pylori OMPs	RP, 4.5 mg protein/mL	*B*	B65660R	1:30
CagA (rec., aa 1-300)	RP, IgG 0.2 mg/mL	*C*	sc-25766	1:10
CagA (rec., aa 748-1015)	RP, IgG 2.0 mg/mL	*D*	HPP-5003-9	1:20
Urease (UreA; rec.)	MM, IgG 0.5 mg/mL	*D*	HPM-5021-5	1:50
VacA (rec., aa 311-819)	RP, IgG 0,7 mg/mL	*D*	HPP-5013-9	1:100
VacA (purified)	RP, whole serum	*E*	123	1:300
NOD1 (rec., aa 494-512)	RP, IgG *	*F*	PAB0266	1:100
E1A/B ligases(rec., aa 41-66)	RP, IgG *	*G*	PW8390	1:50
Polyubiquitinated proteins	MM, FK1 clone, IgM 1 mg/mL	*G*	PW8805	1:30
Mono/polyubiquitinated proteins	MM, FK2 clone, IgG 10 mg/mL	*G*	PW8810	1:10,000
20S proteasome core α/β subunits	RP, IgG *	*G*	PW8155	1:1,000
20S proteasome core α/β subunits	RP, whole serum	*H*	ST1053	1:30
20S proteasome β5i subunit	RP, IgG *	*H*	ST1057	1:300
19S proteasome S2 subunit	RP, IgG *	*H*	539166	1:100
SHP2 tyrosine phosphatase	RP, IgG 0.2 mg/mL	*C*	sc-280	1:10
ERK 1/2 kinases	RP, IgG 0.2 mg/mL	*C*	sc-154	1:10
Ribosomal protein S16	RP, IgG 2 mg/mL	*I*	ab26159	1:5
Cathepsin D	MM, IgG 2.5 mg/mL	*J*	280002	1:10
S-100 protein	RP, IgG 4.1 mg/mL	*K*	Z0311	1:100
Insulin	RP, 14.4 mg/mL	*K*	A0564	1:1,000
Glucagon	RP, IgG 16 mg/mL	*K*	A0565	1:1,000
Negative control for RP	Non-immune rabbit IgG, 0.2 mg/mL	*C*	sc-2027	1:10
Negative control for MM	Non-immune mouse IgG, 0.1 mg/mL	*C*	sc-2025	1:10

OMPs: outer membrane proteins. RP: rabbit polyclonal. MM: mouse monoclonal. rec: recombinant. *, conc. not stated by the manufacturer.
A: Biømeda, Foster City, CA. *B*: Biodesing International, Saco, ME. *C*: Santa Cruz Biotechnology, Santa Cruz, CA. *D*: Austral Biologicals, San Ramon, CA. *E*: Dr. T.L. Cover, Nashville, TN. *F*: Abnova, Taipei City, Taiwan. *G*: BIOMOL International, Plymouth Meeting, PA. *H*: Calbiochem, La Jolla, CA. *I*: Abcam, Cambridge, UK. *J*: Invitrogen, Carlsbad, CA. *K*: Dako, Glostrup, Denmark.
